# Prevalence of macroprolactinaemia in regularly menstruating women with non-toxic goitre or autoimmune thyroid disease

**DOI:** 10.1186/1756-6614-5-20

**Published:** 2012-12-17

**Authors:** Krzysztof C Lewandowski, Danuta Gąsior-Perczak, Aldona Kowalska, Andrzej Lewiński

**Affiliations:** 1 Department of Endocrinology and Metabolic Diseases, Medical University of Lodz, Lodz, Poland; 2 Polish Mother’s Memorial Hospital - Research Institute, Lodz, Poland; 3 Department of Endocrinology and Nuclear Medicine, Holycross Cancer Centre, Kielce, Poland

**Keywords:** Macroprolactin, Thyroid, Goitre, Autoimmune thyroid disease

## Abstract

**Background:**

The so called “big-big” prolactin (Prl), also known as macroprolactin is formed by Prl-immunoglobulin (Prl-IgG) complexes and may cause elevation of serum Prl concentrations measured by standard assays, potentially leading to unnecessary investigations and/or treatment. In our study, we have endeavoured to assess the prevalence of macroprolactinaemia in euthyroid, regularly menstruating women with thyroid disease, as well as to assess whether autoimmune thyroid disease may result in an increased prevalence of macroprolactinaemia.

**Material and methods:**

We measured serum Prl in 182 regularly menstruating women aged 32.7 ± 7.5 years (mean ± SD, range 17–46 years) who attended endocrine clinic either for investigation of non-toxic goitre (n = 86, age 33.2 ± 7.8 years) or with autoimmune thyroid disease (n = 96, age 32.3 ± 7.2 years). Autoimmune thyroid disease was defined as raised titre of at least one anti-thyroid antibody [anti-thyroid peroxidase (anti-TPO), anti-thyroglobulin (anti-Tg) and/or anti-TSH-receptor (anti-TSH-R) antibodies]. All women were clinically and biochemically euthyroid, either without or on treatment with L-thyroxine. In those with raised Prl (i.e., above 530 mIU/l) we ruled out the presence of macroprolactinaemia by polyethylene glycol (PEG) precipitation method.

**Results:**

There was no significant age difference between women with and without autoimmune thyroid disease (p = 0.84). Raised Prl concentrations were found in 10 women with thyroid disease (5.5%), and of those a significant macroprolactinaemia (i.e., reduction of Prl concentrations of more than 60% after PEG precipitation) was found in 9 subjects (4.94%). There were no differences in the prevalence of macroprolactinaemia between women with autoimmune thyroid disease (4 out of 96), and without autoimmune thyroid disease (5 out of 86, p = 0.75).

**Conclusions:**

Approximately one out of twenty women with regular menses is likely to have raised serum Prl that is usually caused by the presence of macroprolactinaemia. Though structure of macroprolactin involves Prl-IgG complexes, there is no evidence that autoimmune thyroid disease is associated with raised prevalence of macroprolactinaemia.

## Background

The so called “big-big” prolactin (Prl), also known as macroprolactin is formed by Prl-immunoglobulin (Prl-IgG) complexes and may cause elevation of serum Prl concentrations measured by standard assays, potentially leading to unnecessary investigations and/or treatment [1]. The term “macroprolactinaemia” is typically applied to cases where concentration of “big-big” Prl exceeds 60% of total serum Prl concentration [2].

It is well proved that autoimmune thyroid disease is often associated with other autoimmune conditions. For instance pituitary antibodies can be observed in about 20% of cases of autoimmune thyroid disease [3]. Furthermore, autoimmune thyroid disease, and Hashimoto thyroiditis in particular, increases the likelihood of fertility problems and/or menstrual irregularities [4,5]. As some studies demonstrated much higher than previously assumed prevalence of partial autoimmune pituitary dysfunction in cases of Hashimoto thyroiditis [6], thus, in our study we have endeavoured to assess the prevalence of macroprolactinaemia in euthyroid, regularly menstruating women with thyroid disease, as well as to assess whether autoimmune thyroid disease *per se* may result in an increased prevalence of macroprolactinaemia.

## Material and methods

We measured serum Prl in 182 regularly menstruating women aged 32.7 ± 7.5 years (mean ± SD, range 17–46 years) who attended endocrine clinic either for investigation of non-toxic goitre (n = 86, age 33.2 ± 7.8 years) or with autoimmune thyroid disease (n = 96, age 32.3 ± 7.2 years). Autoimmune thyroid disease was defined as raised titre of at least one anti-thyroid antibody [anti-thyroid peroxidase (anti-TPO), anti-thyroglobulin (anti-Tg) and/or anti-TSH-receptor (anti-TSH-R) antibodies]). Eighty eight women of this group of patients with autoimmune thyroid disease (91.7%) were diagnosed with Hashimoto thyroiditis, while the remaining eight (8.3%) had a history of Graves’ disease. All women in both groups were clinically and biochemically euthyroid, either without or on treatment with L-thyroxine [where 39 out of 96 patients with autoimmune thyroid disease (40.6%) received L-thyroxine]. None of these patients received any medication that might raise Prl concentrations. Cases of “stress-induced” hyperprolactinaemia were excluded as described by Karasek et al. [7]. In those with genuinely raised Prl (i.e., above 530 mIU/l) we ruled out the presence of macroprolactinaemia by a polyethylene glycol (PEG) precipitation method. This method involves precipitation of monomeric Prl-IgG complex with 25% PEG. Concentration of Prl is assessed before and after PEG precipitation. Significant macroprolactinaemia is said to be present, where Prl recovery in the second sample is less than 40%. According to some authors, 40–60% recovery is considered to represent, the so called “grey zone”, where additional methods for detection of macroprolactinaemia might be sometimes necessary [8,9]. The study has been approved by the Ethics Committee of the Polish Mother’s Memorial Hospital - Research Institute, Lodz, Poland.

## Results

There was no significant age difference between women with and without autoimmune thyroid disease (Wald-Wolfowitz test, p = 0.84). All patients were biochemically euthyroid (TSH – 1.32 ± 0.72 μIU/ml), with no significant difference in TSH concentrations between the investigated groups. Raised Prl concentrations were found in 10 women with thyroid disease (5.5%), and of those a significant macroprolactinaemia (i.e., reduction of Prl concentrations of more than 60% after PEG precipitation) was found in 9 subjects (4.94%) (Table 1). There were no differences in the prevalence of macroprolactinaemia between women with autoimmune thyroid disease [4 out of 96 (4.16%)], and without autoimmune thyroid disease [5 out of 86 (5.8%), p = 0.75, Fisher’s exact test].

**Table 1:** Prevalence of macroprolactinaemia in regularly menstruating women with and without autoimmune thyroid disease; p = 0.75 (non-significant)

	**Autoimmune thyroid disease**		
	**Yes**	**No**	**Together**
Macroprolactinaemia - No	92 [95.8%]	81 [94.2%]	173 [95.05%]
Macroprolactinaemia - Yes	4 [4.2%]	5 [5.8%]	9 [4.95%]
Together	96	86	182 [100%]

## Discussion

To the best of our knowledge, this is the first study on the prevalence of macroprolactinaemia in biochemically euthyroid, regularly menstruating women with non-toxic goitre or autoimmune thyroid disease in Poland. In our study we observed raised Prl concentration in ten out of 182 regularly menstruating women with thyroid disease, where in 9/10 cases (i.e., 4.9% of the total), this was caused by macroprolactinaemia. In a single case, the presence of macroprolactinaemia could not be totally discounted as Prl recovery fell into the “grey zone” of 40–60%. Our data, therefore demonstrate that macroprolactiaemia might be present in about 1/20 of regularly menstruating, clinically and biochemically euthyroid women with various forms of thyroid disease. This observations might have significant clinical relevance, given that women with thyroid disease, particularly in cases of concomitant autoimmune process, are more likely to undergo investigations, e.g., in cases of fertility problems, miscarriages, etc. There are no Polish data on the prevalence of macroprolactinaemia in population of regularly menstruating women, while the data from foreign literature are conflicting. For instance, Bjøro et al. [10] report macroprolactinaemia in only 0.2% of healthy, regularly menstruating women and in 0.02% of healthy men, while other studies report much higher prevalence of macroprolactinaemia. For instance, Hattori et al. [11] report prevalence of macroprolactinaemia in 3.86% of healthy, regularly menstruating women and in 3.13% of healthy men, while according to the recent Hungarian study, the prevalence of macroprolactinaemia was about 1.5% among 131 healthy blood donors (50 men) [12].

In keeping with the data of Kavanagh-Wright et al. [13], we failed to find an increased prevalence of macroprolactinaemia among women with autoimmune thyroid disease. An explanation for these findings may be offered by the reported stimulation in rats of antibodies to Prl, when the animals were injected with phosphorylated Prl usually confined to within the pituitary [14]. The authors speculate that some form of asymptomatic hypophysitis, possibly of viral origin, could be associated with leaking of pituitary phosphorylated Prl into the systemic circulation. The usually sequestered phosphorylated protein might then induce antibodies to Prl, which react with the hormone to form macroprolactin molecules. Thus, this would be a different mechanism from the one involved in generation of thyroid autoantibodies.

In conclusion, we report that approximately one out of 20 euthyroid women with regular menses and either goitre or autoimmune thyroid disease is likely to have raised serum Prl that is almost exclusively caused by the presence of macroprolactinaemia. Though structure of macroprolactin involves Prl-IgG complexes, we have found no evidence that autoimmune thyroid disease is associated with raised prevalence of macroprolactinaemia.

## Competing interests

The authors declare that they have no competing interests.

## Authors’ contributions

KCL designed the study and prepared the manuscript, DG-P was involved in collection of the data and preparation of the manuscript, AK was involved in funding of the study, as well as in supervision of data acquisition, AL was involved in design of the study and in scientific revision of the manuscript. All authors have read and approved the final manuscript.
